# Application of Monolayer Graphene and Its Derivative in Cryo-EM Sample Preparation

**DOI:** 10.3390/ijms22168940

**Published:** 2021-08-19

**Authors:** Ke Wu, Di Wu, Li Zhu, Yi Wu

**Affiliations:** 1 Ministry of Education Key Laboratory of Cell Activities and Stress Adaptations, School of Life Sciences, Lanzhou University, Lanzhou 730000, China; wuk19@lzu.edu.cn (K.W.); dwu15@lzu.edu.cn (D.W.); 2 Electron Microscopy Centre of Lanzhou University, Lanzhou 730000, China; 3 MOE Key Laboratory of Environment and Genes Related to Diseases, School of Basic Medical Sciences, Xi’an Jiaotong University, Xi’an 710049, China

**Keywords:** cryo-EM, single-particle, sample preparation, graphene, graphene oxide, chemical modification

## Abstract

Cryo-electron microscopy (Cryo-EM) has become a routine technology for resolving the structure of biological macromolecules due to the resolution revolution in recent years. The specimens are typically prepared in a very thin layer of vitrified ice suspending in the holes of the perforated amorphous carbon film. However, the samples prepared by directly applying to the conventional support membranes may suffer from partial or complete denaturation caused by sticking to the air–water interface (AWI). With the application in materials, graphene has also been used recently to improve frozen sample preparation instead of a suspended conventional amorphous thin carbon. It has been proven that graphene or graphene oxide and various chemical modifications on its surface can effectively prevent particles from adsorbing to the AWI, which improves the dispersion, adsorbed number, and orientation preference of frozen particles in the ice layer. Their excellent properties and thinner thickness can significantly reduce the background noise, allowing high-resolution three-dimensional reconstructions using a minimum data set.

## 1. Introduction

The cryo-electron microscopy (Cryo-EM) technique began in the early 1980s as samples can be embedded in vitrified ice by plunge freezing which was developed by Jacques Dubochet [1,2,3,4]. In the following four decades, it has gradually developed into a common approach for determining the three-dimensional (3D) structure of various macromolecules and their complexes in near-native states [5,6,7,8,9,10,11,12]. In the early stage, except for highly symmetrical virus particles, which generally attain near-atomic resolution [13], the resolution of other proteins generally does not exceed 7–8 Å [14]. Combined with the X-ray crystal diffraction technique, the high-resolution crystal structures of protein fragments can be docked into the relatively lower-resolution cryo-EM map [15], whereby the overall molecular architecture or the pseudo-atomic model of the macromolecular machine can be determined accordingly. However, since 2013, a resolution revolution has occurred in the field of cryo-EM accompanied by technological innovations, including hardware, such as improved electron microscopes, more stable sample stages, especially direct electron detectors with high detection quantum efficiency [16,17,18], and image processing software, such as the 3D classification procedure in RELION [19], the heterogeneous refinement or 3D variability programs in cryoSPARC [20], which are powerful approaches for separating different conformations in the biochemically purified samples. Cryo-EM now has become a routine technology to obtain 3–4 Å high-resolution structures [21], realizing the reconstruction of an actual atomic model of macromolecules.

Although it has been recently reported that the cryo-EM single-particle technique can achieve a resolution as high as 1.22 Å [22], meanwhile the quality of structure appears better than that obtained at the same level of resolution by X-ray crystal diffraction [23]; this may not be universally achievable. It mostly depends on the property of the macromolecule itself, such as the intrinsic stability and its behavior in the specimen preparation. Some types of particles tend to absorb to the air–water interface (AWI) in a preferred orientation or into aggregates [24]; some complexes assembled from multiple protein components may become disassociated, while others may partially or even completely denature at such interfaces during the very fast plunge-freezing. In other cases, some proteins prefer to stick to the hole edge of the grid or be squeezed there due to the very thin ice thickness in the center of the hole. Therefore, sample preparation and corresponding optimization seem to be the most critical or rate-limiting points to enable high-resolution structural analysis [25]. To alleviate the above situations, a thin layer of continuous amorphous carbon is usually deposited on the holey substrate of a standard cryo-EM grid to keep the particles away from the AWI and for better particle distribution. Recently, a more popular support substrate, monolayer graphene and its derivative graphene oxide (GO), have been used to replace the conventional thin amorphous carbon film to further ameliorate the sample preparation and the achievable resolution [10,26,27,28]. In this review, we mainly focus on the characteristics of graphene and GO, the recently proposed preparation procedure of graphene or GO suspended EM grid, the derivative surface modifications, and the related benefits to cryo-EM sample preparation.

## 2. Supporting Films of Amorphous Carbon and Monolayer Graphene

### 2.1. Amorphous Carbon Film and Its Limitations

The commonly used cryo-EM grid for better automatic data acquisition is a perforated carbon support foil with pre-defined hole size, shape, and regular arrangement, such as Quantifoil grids. One can also use a homemade carbon-coated holey grid with variable hole sizes and bar width. Ordinarily, the coated holey amorphous carbon film is not flat and has a non-uniform thickness [29]. Its surface may be contaminated by organic substances during the production, leading the hydrophilicity to vary from batch to batch or even from grid to grid in the same package. These may result in a variable ice thickness within a single grid or even the same mesh, and a region-dependent defocus. Thick ice adds more noise and requires a larger defocus value to enable a sufficient contrast for particle-picking and the subsequent processing, leading to the information lost in the high spatial frequency. If the ice layer is too thin, it will push the particles to the edges of holes, which may cause deformation of the particles under the high surface tension [30]. A smaller region with suitable thickness of the ice layer inevitably reduces the effective observation area for data collection. Besides, the parameters of glow-discharging for hydrophilization need to be constantly optimized. Poor reproducibility in sample preparation due to supporting grid also results in the waste of the sample and electron microscope time.

Meanwhile, although amorphous carbon films have the semiconducting property, their electrical conductivity and mechanical strength can be largely decreased due to the manufacturing process [31]. When the specimen at the liquid nitrogen temperature interacts with the high-energy electron beam, a positive charge accumulates due to the insufficient conductivity of the holey carbon films, which is thought a probable contributor to the beam-induced motion [32] and the radiation-induced sample damage [9]. Deformation ascribed to the insufficient mechanical strength may also increase the beam-induced motion [33], leading to image blurring and loss of high-resolution information.

**Figure 1:**
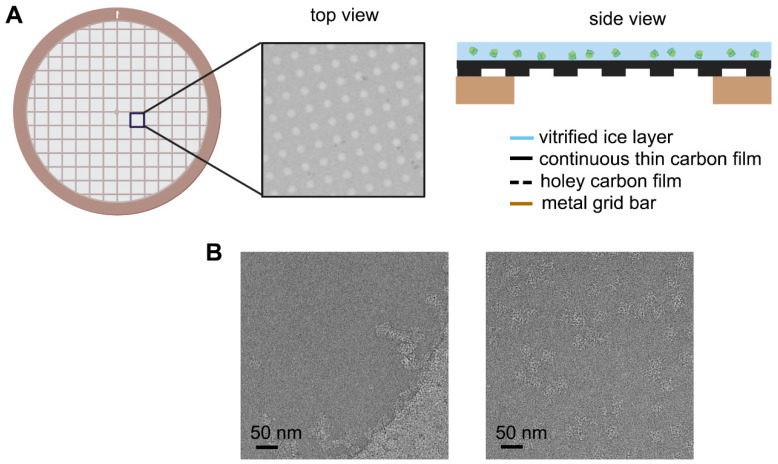
Schematic diagram of improved EM grid and its improvement on the particle distribution. (**A**) Commonly used arrayed EM grid covered with additional layer of continuous thin carbon film on the holey carbon film. (**B**) Example of ryanodine receptor 1 (RyR1) distribution in the vitrified ice without (left panel) and with (right panel) continuous thin carbon film spanning on the hole.

Usually, appending an additional ultra-thin carbon layer above the holey carbon film can primarily circumvent the uneven ice thickness caused by the conventional EM grid (Figure 1A) and improve the dispersability and orientation distribution of particles in the ice (Figure 1B) [34]. It can also somehow increase the stability of the sample under electron beam radiation [35]. However, despite providing adequate mechanical strength to support the ice layer, the newly added amorphous carbon layer, usually with a thickness of 20–50 Å [36], inevitably increases the background noise, limiting its use to relatively large molecules and making it challenging to achieve high resolution even with an ideal ice layer, especially for small protein molecules.

### 2.2. Properties of Monolayer Graphene

As a novel and promising material, graphene emerged for developing nanocomposites around 2010 [37,38]. The first exfoliation of graphene was achieved by Andre Geim and Konstantin Novoselov in 2004 [39], which was awarded the Nobel Prize in Physics in 2010. Before that, no monolayer 2D crystalline material had ever been successfully generated. The deposits obtained by chemical exfoliation of graphite were multilayer flakes. Many crystals exist as flake stacks, with atoms bonded by solid forces within each layer, while only weak van der Waals forces exist between the layers. However, when researchers attempt to peel them off into single-atom layers, these crystals become unstable due to the reduced number of layers, which in turn disintegrate. Apart from the thermodynamic instability, the main reason why 2D crystals were not discovered previously is that single-molecule films are completely transparent in visible light, and not visible with optical microscopy on commonly used substrates (e.g., glass), and have no clear signal under transmission electron microscopy [40].

Unlike other sheet crystals, monolayer graphene can still exhibit a relatively high crystalline quality and continuity even in the form of single atomic layers. Graphene is a hexagonally arranged honeycomb-like planar sheet of carbon atoms with adjacent carbon atom spacing of about 0.142 nm, rich in σ-bonds within its planar surface. These σ-bonds contribute to most of the structural stability of graphene. Some unhybridized π-bonds are also prominent outside the graphene carbon planes, which determine graphene’s electrical, thermal, and optical properties [41]. Due to its excellent properties such as flat and continuous surface, unexpected mechanical strength [42], high thermodynamic stability and chemical inertness [43] as well as outstanding semimetallicity [44,45], monolayer graphene has been involved in many scientific fields since its discovery.

## 3. Preparation of Grid Covered with Graphene or Graphene Oxide Film

Besides its good mechanical strength and electrical conductivity, monolayer graphene with only 0.34 nm thickness can also significantly minimize electron scattering in transmission electron microscopy. With all these favorable properties, graphene has been applied to cryo-EM as a sample support film to replace the amorphous carbon film, which has already achieved some success and important breakthroughs [10,46]. However, monolayer graphene has not been widely adopted in the field of cryo-EM for biological samples. There are mainly two reasons for this: (1) the surface of graphene is hydrophobic, resisting proteins adsorption, and is susceptible to contamination during production. The commonly used plasma cleaner for glow-discharging should be operated very carefully, or a specific recommended brand and model may be used; otherwise, the integrity will be easily destroyed because of its super thin thickness. (2) Except for the commercially purchased products, most monolayer graphene-covered EM grids are prepared by researchers themselves. Home-made graphene grids usually have low coverage of single layer and poor reproducibility, significantly decreasing the success rate of sample preparation or data acquisition efficiency. Thus, obtaining monolayer graphene-covered grids with high coverage, clean and hydrophilic surface has become a primary issue in sample preparation. New methods for the preparation of graphene-covered grids have been developed, and the quality has been improved. Herein, we briefly describe several graphene-covered grid preparation methods and compare the differences between them.

### 3.1. Graphene Grid Fabrication

The preparation of graphene-covered grid mainly contains two steps: obtain the high-quality monolayer graphene and transfer it to the grid which is already coated with a holey film made of amorphous carbon or other materials [47]. Since the first successful exfoliation from the surface of a piece of graphite using sticky tape, there have been many fabrication methods developed to obtain single-layer graphene, including mechanical exfoliation from graphite [48,49], epitaxial growth on the surface of silicon carbide crystals or metal substrate [50,51,52], chemical exfoliation (chemical oxidation of graphite and subsequent reduction of the exfoliated graphite oxide sheets) [53], and chemical vapor deposition (CVD) [51,54,55,56]. The CVD method is widely adopted with various advantages, such as excellent reproducibility, homogeneity, and crystalline quality. After limited graphene growth on Ni by CVD with success [57], graphene growth on copper-foil substrates emerged as an alternate route for large-area synthesis with higher monolayer coverage [51,54].

**Figure 2:**
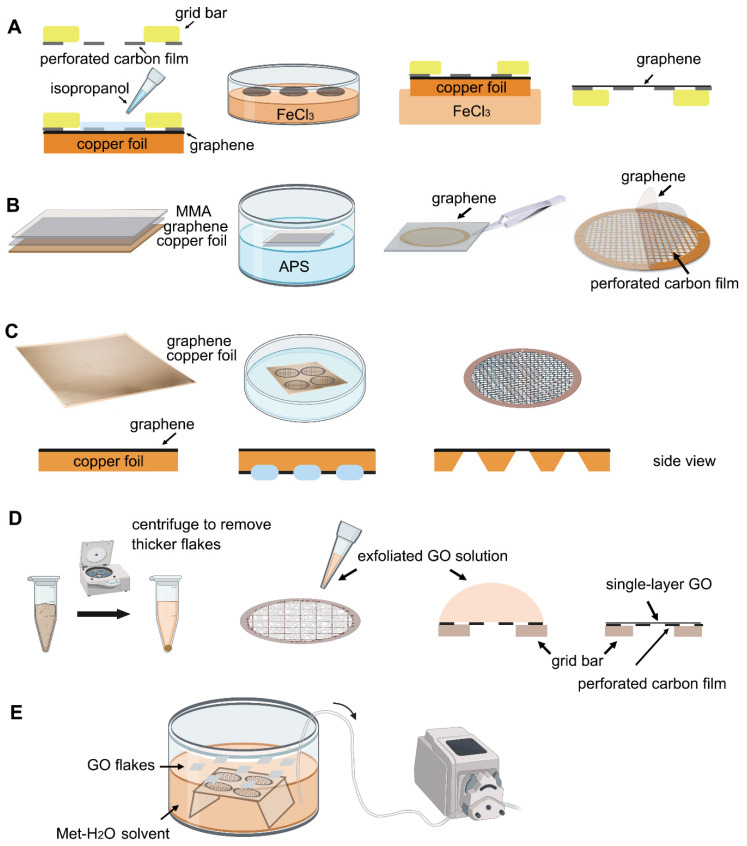
Schematic representation of different graphene grid (**A**–**C**) and graphene oxide grid (**D**,**E**) fabrication methods in primary steps. (**A**) The method uses isopropanol for adhering perforated carbon film with graphene by solvent wetting and then floats the grid-graphene-copper sandwich in FeCl_3_ to etch away the copper substrate [34]. (**B**) The method uses MMA to transfer graphene to the APS solution. After transferring graphene to the EM grid, the MMA film can be digested [58]. (**C**) The method directly etches the backside of copper foil that supports graphene growth into arrayed Cu grids by direct photolithography in batch fabrication [59]. (**D**) The method centrifuges the GO flakes to make them more homogeneous, then apply a droplet of GO solution to the perforated carbon film. After waiting for the interaction between GO flakes and the holey carbon, blot away the excess solution and let the GO-covered grid dry for use [26]. (**E**) Use methanol-water (Met-H_2_O) solvent to disperse GO flakes, embed the grids with perforated carbon film facing up, and slowly pump out the solution to let the GO flakes adhere to the submerged grids [61].

**Figure 3:**
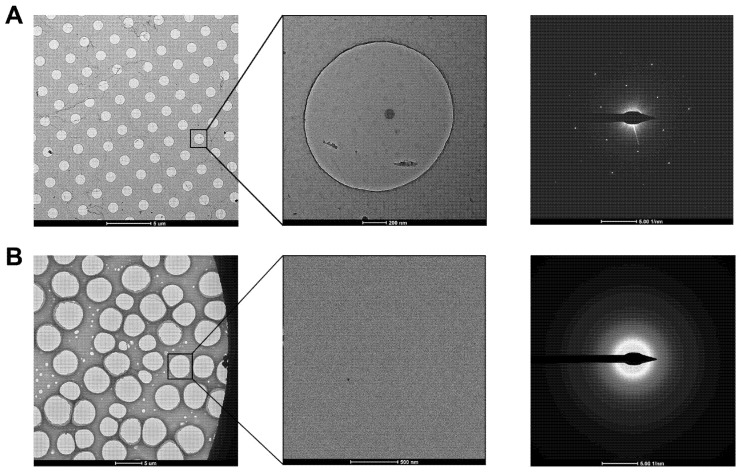
Examination of graphene and GO grids in the diffraction mode. (**A**) Graphene grid prepared using Quantifoil grid. The diffraction pattern showed the single-layer coverage in the zoomed-in hole. (**B**) GO grid prepared using holey carbon grid (purchased from Beijing Zhongjingkeyi Technology Co., Ltd. Beijing, China). The diffraction pattern showed the multi-layer coverage in the zoomed-in hole.

Transferring monolayer graphene to the perforated carbon foil with high coverage is the technical bottleneck for its cryo-EM application. Graphene grown on the copper foil by CVD, which can also be purchased from Graphene Supermarket, is often used to prepare the graphene grid. One of the methods reported in 2016 uses isopropanol for adhering perforated carbon film (Quantifoil Au 300 1.2/1.3) on the gold grid to graphene by solvent wetting, and then floats the grid-graphene-copper sandwich in FeCl_3_ to etch away the copper substrate (Figure 2A) [34]. Another method reported in 2019 uses a thin layer of methyl methacrylate (MMA) film to transfer graphene to the ammonium persulfate (APS) solution to dissolve the copper substrate. After washing the MMA-graphene film in deionized (DI) water, it is transferred to the grid (Quantifoil Au 300 1.2/1.3). Finally, the MMA film is digested with acetone and isopropanol to obtain the graphene-covered grid (Figure 2B). The entire preparation process takes about a few hours and requires no other special equipment. As reported, up to 99% monolayer graphene coverage enables more than 70% of grid area to be useful for data collection (Figure 3A). The researchers used ozone to treat the graphene gently to make it hydrophilic and reached 2.6 Å resolution for streptavidin [58], demonstrating that this method effectively helps small molecules to achieve the high-resolution reconstruction. However, in 2020, researchers directly etched Cu foil’s backside that supports graphene growth into arrayed Cu grids by direct photolithography in batch fabrication (Figure 2C). The process benefits from being transfer-free and polymer-free, thus generates ultraclean single-layer graphene film [59]. After that, an experimental design reported in 2021 avoids the step of hydrophilizing graphene. It uses a support floatation block holding up to 10 µL in each well to directly transfer samples to graphene film after a short incubation [60]. The graphene grid preparation procedure is modified using isopropanol to adhere graphene and carbon and FeCl_3_ to etch Cu foil.

### 3.2. Hydrophilization of Graphene Surface

Even though graphene has many outstanding properties, it should first be made hydrophilic to be usable for biological samples. Besides directly transferring samples to the graphene in the solution [60], an UV/ozone cleaner (UVOCST 10 × 10 system) generating 185 nm and 254 nm UV light can be used for a mild modification of graphene’s surface, making it hydrophilic [58]. A low-energy hydrogen plasma treatment can also be considered. Typical transfer methods may cause contamination of graphene with adsorbents. A low-energy and pure hydrogen plasma (Fischione 1070 with a Dominik Hunter model 20H-MD hydrogen generator [34]) can convert graphene to its fully hydrogenated form without destroying its lattice. A 30 s processing time can remarkably remove the contamination and decrease the background noise. With an increasing exposure time, the air–water–graphene contact angle decreases exponentially, in positive proportion to the hydrophilicity [62]. Besides, an oxygen-plasma controlled with a low-energy power of 40 W and a flow rate of 5 sccm oxygen can also be used to treat the graphene. By optimizing the treatment time, the desirable wettability of graphene grid can be obtained [59]. Notably, after processing, graphene should be used immediately to avoid the accumulation of contamination or any breakage.

### 3.3. Graphene Oxide Grid Fabrication

Graphene oxide (GO) is a hydrophilic derivative of graphene and is thus considered a near-ideal material for cryo-EM sample preparation. It can be exfoliated from graphite oxide (produced according to the Hummers method [63]) by sonication at 35 kHz for 30–60 min. Then leave the suspension overnight or centrifuge at low-speed (~980 to 6000 g) to further precipitate thicker graphene/graphite flakes [26,64,65]. It is essential to control the concentration of exfoliated graphene oxide flakes, otherwise folded layers may accumulate. A storage time that is greater than 2–3 weeks may result in wrinkling or polymerization of the exfoliated graphene oxide, affecting its application [26,64].

The next step is to transfer the GO single-layer flakes to EM grid. One of the methods allows directly applying a 4 μL drop of exfoliated GO solution to the glow discharged (using H_2_ and O_2_ mixed gas) EM grids with ~1 μm hole size for ~1 min (drop-casting) [26]. The GO can be absorbed to the holey carbon film through electrostatic interaction [64] and then be held in place by van der Waals forces [51]. The excess water is removed by filter paper and the grids are dried under nitrogen gas to minimize contamination (Figure 2D). Before use, amorphous material can be removed, and the properties of graphene can be optimized by baking at 300 °C for 3–5 min [26]. For convenience, a commercial suspension of monolayer GO flakes (e.g., Sigma-Aldrich) can be used. However, even with bath sonication and the following centrifugation to remove the possible aggregated flakes due to the storage age, the coverage uniformity of prepared grids covered with single-layer GO is not satisfied (Figure 3B). It requires extensive screening to find good fabrication [61]. Therefore, an improved fabricating method with higher repeatability and ideal GO coverage has been proposed. The methanol-dispersed GO flakes solution is mixed into pure water, which can help GO flakes spread and enrich easily on the air–water interface to assemble into a continuous thin film. A peristaltic pump is then used to pump out the water slowly, resulting in the GO film finally adhere to the submerged grids with holey carbon film facing up (Figure 2E) [61]. It is further confirmed that the Met-H_2_O in a 5:1 ratio helps larger monolayer GO flakes deposit on the EM grids, which also facilitate to reduce the preferred orientation of protein samples in cryo-EM specimen preparation [65,66].

## 4. Advantages on Single-Particle Cryo-EM Sample Preparation

### 4.1. Improvement on Protein Dispersion and Orientation

**Figure 4:**
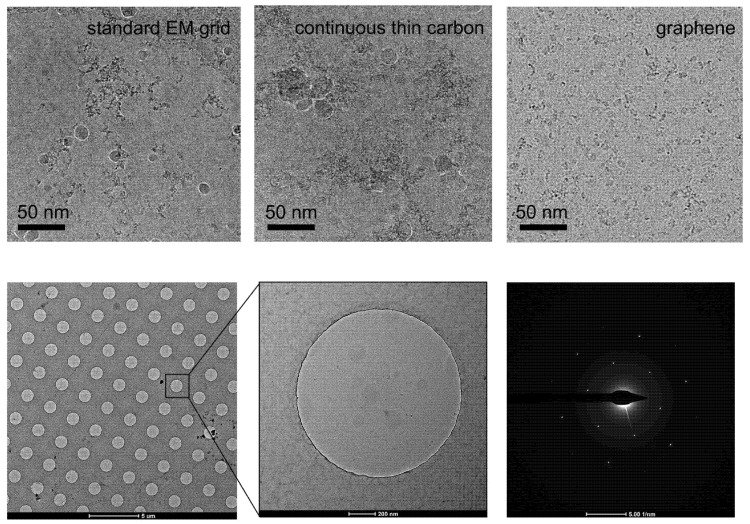
Improvement of graphene grid on the protein distribution by preventing from adsorption to the AWI. The graphene grid examined in the diffraction mode before sample preparation showed single-layer coverage.

Depending on the property of the individual protein sample, the behavior in the suspended ice layer varies from protein to protein. Some proteins tend to favorably adsorb on the carbon film rather than distribute in the vitreous ice spanning the holes [67,68]. In this case, only when the carbon film is saturated with adsorbed protein will the remaining protein molecules be pushed into the holes. A possible solution is to increase the sample concentration. However, to some challenging targets, the particles may get aggregated other than dispensing evenly in the holes at the high concentration level. Graphene or GO covered holey carbon films have been proved to significantly help particles monodisperse in the holes [58,65,69] with a concentration just a little higher than that needed for negative staining sample preparation, which will dramatically save samples (Figure 4). Furthermore, when use low-energy hydrogen plasma to treat the appended graphene film, the number of particles adsorbed increases in a dose-dependent mode, thereby allowing the use of graphene to tune particle distribution [62]. Additionally, the GO covered grids fabricated by applying flakes suspended in the modified Met-H_2_O mixture enable to enrich particles by adsorption, which is meaningful for samples that are challenging to obtain. More importantly, it may also help to resolve the preferred orientation problem [61,65,66].

### 4.2. Prevention of Protein Denaturation Caused by Air–Water Interface

For achieving a high-resolution structure, the vitreous ice in which biological molecules are embedded should be evenly thin to give better contrast and hence a lower defocus range for data acquisition. As a result, the molecules are inevitably exposed to the air at a high surface-to-volume ratio. The air–water interface (AWI) has been considered unfavorable for proteins to maintain their native conformation [70,71,72,73]. Protein denaturation due to the AWI can happen within milliseconds or even less [71,72,74,75,76], which can be summarized in three stages [77]: first, protein adsorbs to the AWI, followed by shearing stress disrupts the protein structure and the polypeptide chains unfolding to form inactive aggregates [78]. Then, the denatured protein molecules trigger the aggregation of more denaturing proteins, which may also lead to the preferred orientation even a two-dimensional array can be observed [79].

To avoid the adverse effects of the AWI on proteins, the following precautions can be considered [77]: (1) stabilizing the buffer conditions by adding stabilizer, such as glycerol or trehalose, or optimizing the buffer composition, such as pH and ionic strength, may also solve the preferred orientation; (2) shielding the AWI with surfactants to prevent protein adsorption [80]; (3) rapidly applying samples and plunge-freezing the proteins before they adsorb to the AWI and denaturation; and (4) allowing the proteins to adsorb to a structure-friendly support substrate to avoid contacting the AWI. In short, the last one is the most feasible. The addition of stabilizers and surfactants can diminish the signal-to-noise ratio (SNR) dramatically, especially disastrous for the small molecules, and change the surface tension of the solution and thus the sample preparation conditions. In addition, there is no guarantee that the sample preparation can be faster than the adsorption of proteins to AWI and the subsequent denaturation. Although an amorphous carbon film will undoubtedly reduce the SNR, the background noise from the graphene or GO film is negligible [24]. Cryo-electron tomography (Cryo-ET) technology can be used to examine the particle distribution along the z-direction of the vitrified ice. Fan et al. used the graphene grid to challenge the 52 kDa streptavidin reconstruction. Interestingly, they found that more than 80% of particles stuck to the graphene-water interface (GWI) contributed to the high-resolution reconstruction, despite uneven distribution compared to those adsorbed to the AWI [10]. Graphene grids can also enable a high-resolution reconstruction using a relatively small data set. Joppe et al. used only 15,000 particles of yeast fatty acid synthase to achieve a 3.1 Å resolution map [46]. It was also found that applying protein solution on the backside of the EM grid, which is opposite to the graphene-carbon coated side, will effectively keep proteins from denaturation [24]. Generally, by adding graphene or GO film to the EM grids, proteins tend to adsorb at the GWI, avoiding denaturation and further formation of larger denatured protein oligomers due to adsorption at AWI, and facilitating a reduction in the number of particles required to reconstruct a high-resolution structure.

### 4.3. Other Modifications of Graphene Surface in Sample Preparation

Despite the many excellent properties of graphene and GO support films in frozen sample preparation, some other chemical modifications on their surfaces have been designed to further solve particular problems encountered even with these films applied. Naydenova et al. developed a low-energy plasma surface modification system to allow different covalent functionalizations (thiol-, amylamine/hydrogen-, amine- and phenol-functionalized) across the graphene layer on a single gold grid [36]. The regionally functionalized graphene-gold grid will help quickly find the optimal surface conditions for frozen sample preparation with the least amount of work. Additionally, it can significantly reduce specimen movement during imaging, minimize orientation bias, improve the image quality, allow a high-resolution structure built using a minimal data set. Unlike this method, Wang et al. modified the GO surface using amino groups with a PEG spacer between them [66,81]. The Amino-PEG-GO grids can concentrate particles with better distribution and orientation. Notably, the PEG spacer can keep particles away from both the GO-water interface and the AWI, further protecting the sample from potential denaturation and aggregation. Besides, Nα, Nα-dicarboxymethyllysine (NTA) has also been linked to the GO sheets [82]. After blocking non-selective binding sites between GO-NTA film and non-target proteins, 6× His-tag proteins can be captured directly from cell lysates to the modified supporting film, enabling a breakthrough in the 3D reconstruction of less homogeneous protein samples. A similar Ni-NTA modified functionalized graphene membrane (FGM) grid was also proposed to immobilize His-tagged proteins and complexes hence prevent from partial denaturation caused by adsorbing to the AWI [29], using polymer-free transferred graphene as the supporting substrate [83]. The covalently linked groups add only 1–2 nm thickness compared to the traditional amorphous carbon film (~5 nm), with significantly lower background noise added. Moreover, the FGM is hydrophilic and exhibits a high affinity for His-tagged proteins, which can also act as an affinity filter for proteins without His-tag, simplifying protein purification. Taken together, while maintaining the single-crystal structure and basic properties of graphene, different surface modifications on graphene or GO film can further help to adjust the type, quantity, distribution, and orientation of the adsorbed proteins, keeping them away from denaturation due to potentially stick to the AWI.

## 5. Conclusions

Although the graphene or GO support has many excellent properties, whether to use them depends on the target protein or complex. Many specimens exhibiting stability and that monodisperse in the ice layer have been reconstructed at a high resolution just using regular perforated amorphous carbon film. However, for those problematic specimens that are prone to sticking to the AWI or poor distribution in the holes, the developed graphene or GO film suspended over holes could be the optimal and unique solution with minimal background noise added. In graphene/GO film fabrication, the single-layer, high coverage, easy to operate, and reproducibility should be the most considerable aspects. A higher monolayer coverage is the most crucial factor to the small proteins with molecular weight lower than 100 kDa. Compared with graphene films, GO films may be more widely used because they are naturally hydrophilic and do not require additional hydrophilization, a step that is very important for naturally hydrophobic graphene films. For graphene films, incomplete hydrophilization may affect the uniformity of ice thickness as well as particle dispersion. In addition, whether additional expensive equipment is needed may also be a consideration for the local implementation for both graphene and GO films.

For some proteins, adding graphene or GO membrane alone cannot prevent their adsorption to AWI. Thus, it may be required for specific chemical modification on the graphene or GO membrane surface to help the particles completely avoid denaturation or aggregation. In addition, changing the grid material, such as replacing the copper mesh with a gold mesh or replacing the amorphous carbon film-covered gold mesh with a gold foil-covered gold mesh, may be more helpful to reduce the sample movement and further improve the resolution.

## Data Availability

No new data were created or analyzed in this study. Data sharing is not applicable to this article.
